# E-cigarette puff topography instruction to enhance switching among COPD patients who smoke

**DOI:** 10.3389/fpubh.2025.1664400

**Published:** 2025-10-02

**Authors:** Eleanor L. S. Leavens, Theodore L. Wagener, Leah Lambart, Matthew S. Mayo, Lexie Brown, Edward F. Ellerbeck, Sandra A. Billinger, Branden Comfort, Jennifer Woodward, Brent Sear, Spencer Beaman, Lisa Sanderson Cox, Nicole L. Nollen

**Affiliations:** 1 Department of Population Health, University of Kansas School of Medicine, Kansas City, KS, United States; 2 University of Kansas Comprehensive Cancer Center, Kansas City, KS, United States; 3 Department of Internal Medicine, The Ohio State University, Columbus, OH, United States; 4 The Ohio State University Comprehensive Cancer Center, Columbus, OH, United States; 5 Department of Biostatistics and Data Science, University of Kansas Medical Center, Kansas City, KS, United States; 6 Department of Neurology, University of Kansas Medical Center, Kansas City, KS, United States; 7 Department of Internal Medicine, Division of General and Hospital Medicine, University of Kansas Medical Center, Kansas City, KS, United States; 8 Department of Family Medicine, Family Medicine and Community Health, University of Kansas Medical Center, Kansas City, KS, United States; 9 Clinical Pharmacology Shared Resource, University of Kansas Cancer Center, Kansas City, KS, United States

**Keywords:** smoking, COPD, tobacco harm reduction, topography, e-cigarette

## Abstract

**Introduction:**

Patients suffering from chronic obstructive pulmonary disease (COPD) who smoke often face significant challenges when attempting to quit. However, switching to less harmful alternatives such as electronic cigarettes (e-cigarettes) may help mitigate tobacco-related health outcomes. Training COPD patients who smoke to adjust their puffing topography could enhance nicotine delivery and satisfaction, thereby facilitating their transition to less harmful alternatives. This pilot study examined a novel puffing topography feedback intervention to facilitate switching to e-cigarettes among COPD patients.

**Methods:**

The study participants (*N* = 46) completed a 12-week e-cigarette switching trial in which they were randomized (1, 1:1) to (1) brief advice, (2) low-intensity, or (3) high-intensity topography training. This approach differed in the extent to which participants took longer puffs to maximize nicotine delivery, alleviate craving and withdrawal symptoms, and facilitate switching. Lab-based vaping sessions were conducted at weeks 0 (visits 1 and 2; separated by 48 h) and 12 (visit 3) to assess changes in puff duration (primary outcome), craving, withdrawal symptoms, and nicotine delivery. Effect size estimates are presented.

**Results:**

Puff duration was similar across conditions at baseline (range: 1.14–1.70s), and contrary to the hypotheses, neither low- nor high-intensity training led to meaningful changes in puff duration over time compared to brief advice (Hedge’s g = 0.34). While the effects were minimal, the brief advice condition demonstrated the highest rate of complete switching (38.5%) and the largest reduction in cigarette smoking (*M∆* = −17.6, *SD* = 10.0; Hedge’s g = 0.78) across treatment groups.

**Discussion:**

E-cigarettes exhibit high potential to minimize harm in COPD patients who smoke. However, puff topography training did not alter switch success or reduction in cigarette smoking as compared to the brief advice to switch.

**Clinical trial registration:**

## Introduction

Tobacco use continues to be the leading cause of preventable death and disease in the US (1). In 2022, approximately 28.8 million US adults reported current cigarette smoking (2). Tobacco use is the primary risk factor for chronic obstructive pulmonary disease (COPD) and accounts for over 70% of cases in the US (3). COPD is a chronic lung condition, which is caused by damage to the lungs, resulting in inflammation and irritation that restricts airflow, as well as causing difficulty in breathing and significantly diminished quality of life (4). For COPD patients who smoke, the first step toward improving health is smoking cessation (5–7). While approximately 90% of COPD patients who smoke report interest in quitting (8, 9), only 5–10% are successful, despite using FDA-approved pharmacotherapy (10). There is a critical need to identify strategies to reduce combustible cigarettes exposure in this priority population whose disease is caused and exacerbated by cigarette smoking, yet face particular difficulty in quitting.

Tobacco harm reduction, or transitioning adults who smoke to less harmful products such as electronic cigarettes (e-cigarettes), is a strategy that results in reduced exposure to tobacco-related toxicants and may result in improved health outcomes relative to the continued use of combustible cigarettes (11–13). This strategy is particularly promising for adults who smoke and are unable or unwilling to quit smoking and would otherwise continue smoking combustible cigarettes. E-cigarettes, when used consistently, are effective for smoking cessation (14). However, the extent of harm reduction is directly related to the degree of switching or the reduction in cigarette smoking (12, 15). Most adults who smoke and transition to e-cigarettes make a partial switch and transition to a pattern of dual e-cigarette and combustible cigarette use (13, 16). While dual users experience harm reduction benefits relative to continued exclusive smoking, the benefit is less than that of those who completely switch (12, 15).

One primary factor that predicts successful switching is e-cigarette puff behavior (17, 18). Similar to nicotine replacement therapy (NRT), e-cigarettes aim to replace nicotine from combustible cigarettes, thereby reducing craving and withdrawal symptoms and assisting in the transition. When using NRT, guidelines instruct providers to advise individuals how best to use the product to achieve smoking abstinence. However, those attempting to transition to e-cigarettes are not provided instructions, resulting in a learning curve to obtain sufficient nicotine delivery and reinforcement to achieve cigarette abstinence (18, 19). To this point, cross-sectional studies indicate that exclusive e-cigarette users extract more nicotine from e-cigarettes through longer puffs to effectively reduce their nicotine craving and withdrawal symptoms and facilitate a complete switch compared to naïve or dual users (19–21), indicating that these established users have learned to effectively lengthen their puffs to extract more nicotine. Related studies suggest that puff duration is a key factor in predicting success at switching. Specifically, in a single-arm, 2-week e-cigarette switching study where participants achieved an 80% reduction in cigarettes per day, cigarette reduction was directly related to a corresponding increase in puff duration over time (17). As individuals learned to take longer puffs, they reduced their cigarette smoking.

Given established links between puff duration and successful switching (13, 14) and a critical need to identify strategies to reduce exposure to combustible cigarettes in COPD patients, the current study aimed to test a novel puff topography feedback intervention among COPD patients who smoke and assess the impact on puff duration, nicotine delivery, craving and withdrawal symptoms, switching patterns, and cigarette smoking. We hypothesized that higher training intensity would be associated with a significant increase in puff duration, nicotine delivery, a decrease in craving and withdrawal symptoms, an elevated rate of complete switching, and a reduction in cigarette smoking.

## Methods

### Participants

Participants were recruited from the Kansas City, KS, United States and Kansas City, MO, United States. Patients with COPD who were at least 21 years old, spoke and understood English, smoked more than 25 out of the last 30 days for the previous 3 months, had tried but failed to quit smoking in the previous year, were unwilling to make a pharmacotherapy-assisted quit attempt in the next 30 days, and were interested in trying e-cigarettes were eligible. Participants were excluded if they reported using tobacco products other than cigarettes on more than 10 of the past 30 days, reported current use of cessation medications, were pregnant, planning to become pregnant, or breastfeeding, had a cardiovascular or pulmonary event in the past 3 months, reported weekly use of an e-cigarette in the past 6 months or any e-cigarette use in the past 30 days, or had a household member currently or previously enrolled in the study.

### Procedures

**Figure 1:**
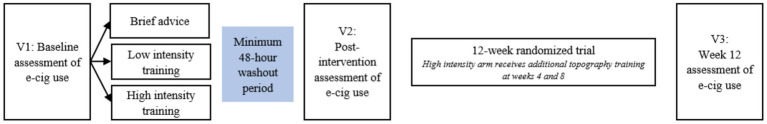
Study flow diagram.

**Figure 2:**
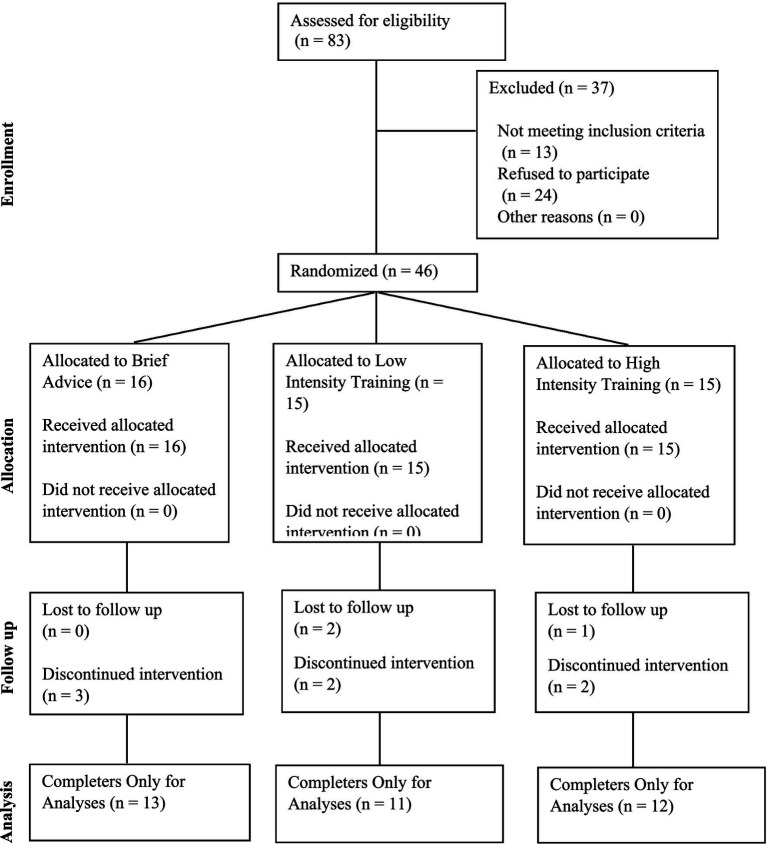
CONSORT participant flow diagram.

We recruited participants from February 2022 to April 2023 through flyers, letters sent on behalf of primary care physicians, and participants from prior smoking cessation studies. All study procedures were reviewed and approved by the University of Kansas Medical Center Institutional Review Board (Figure 1). Participants completed preliminary screening online or by phone to determine initial eligibility. Eligible participants were then invited to complete the final in-person screening and enrollment visit, which consisted of exhaled carbon monoxide [eCO > 10 ppm (22)] to confirm smoking status and a negative pregnancy test for women of reproductive age. Following confirmation of eligibility, participants completed informed consent and baseline measures. Participants sampled two pod-based e-cigarettes (the closed system Vuse Alto and the refillable Evolv Reflex) and two e-liquid flavors (menthol and tobacco) to establish device type and e-liquid flavor preferences. Both devices were used with a 5% nicotine salt-based e-liquid, and both menthol and tobacco flavors were available for both devices. See Figure 2 for CONSORT Flow Diagram.

#### Laboratory methods

Participants completed three lab-based e-cigarette use assessment visits throughout the study, two at week 0 (i.e., lab V1 and V2; conducted within 7 days, with V1 on day 1 and V2 occurring no sooner than day 3 and no later than day 7) and one at week 12 (i.e., lab V3). At the start of each visit, participants were 12-h abstinent from nicotine/tobacco, verified by exhaled carbon monoxide (eCO) (≥50% reduction from final screening) (23). V1 provided estimates of baseline puff topography and resulting nicotine levels from ad libitum e-cigarette use prior to any intervention/training. V2 used the same laboratory methods as V1 and provided estimates of acute changes in puff duration, craving, withdrawal symptoms, and nicotine delivery in the 48-h period between V1 (prior to training) and V2 (followed a single session of brief advice and a single session of topography training). Methods for all lab-based e-cigarette use assessment visits were identical. V1 and V2 were separated by a standard 48-h washout period. Participants were provided with their preferred e-cigarette and e-liquid flavor and engaged in a 30-min ad libitum vaping session. Puff topography was measured throughout the session, and blood samples for nicotine measurement were collected pre- and post-vaping via venipuncture draw.

Following ad libitum e-cigarette use at V1, participants were randomized 1:1:1 to one of the three interventions. Training was provided immediately following ad libitum vaping at V1.

#### Topography training/intervention

*Brief Advice (V1 at week 0):* Participants were given brief advice to switch completely to the study e-cigarette, brief information regarding the relative harm of e-cigarettes compared to combustible cigarette smoking, and basic instructions on the use of e-cigarette.

*Low Intensity Training (V1 at week 0):* In addition to the instructions provided i the brief advice condition, those in the low-intensity training condition underwent a single training session during which they received real-time feedback on their topography. During the session, participants puffed on an e-cigarette connected to the eTop topography device, allowing them to view their puff patterns as they puffed. Trained research staff provided feedback on increasing puff duration to approximately 2 s, and fidelity to this mark was monitored. The training session lasted approximately 30 min.

*High-intensity Training (V1 and V2 at week 0 and weeks 4 and 8):* Participants received the same training as those of the low-intensity training condition at V1. The low- and high-intensity training conditions received the same intervention at V1 and were therefore collapsed for analyses, examining differences from V1 to V2 (see data analysis plan). High-intensity training also received booster topography training sessions (identical to that provided at V1) at week 4 and 8 follow-up visits to provide additional assistance in achieving optimal puff duration to support a complete switch. This condition mimicked the level of support provided to adults who smoke, making a quit attempt using traditional quit smoking strategies, and was included to determine the level of support necessary to achieve a pattern of predominant e-cigarette use.

#### Randomized switching trial methods

At the end of V2 (end of week 0), participants entered directly into the 12-week pilot switching trial. Participants were provided with their preferred e-cigarette and enough e-liquid in their preferred flavor to last until follow-up sessions at weeks 4, 8, and 12 (primary trial endpoint). Study participants were provided with e-cigarettes and e-liquid at no cost. Participants were encouraged to use the e-cigarette frequently and before cravings or withdrawal symptoms occurred. Follow-up visits included eCO measurement, survey completion, and, for the high-intensity training condition only, additional topography intervention at weeks 4 and 8. Participants were compensated $210 for completing all study procedures and assessments.

### Measures

*Participant characteristics:* The baseline participant characteristics included age, sex, race/ethnicity, educational attainment, income, home ownership, and marital status.

*Baseline smoking characteristics*. Baseline smoking history included cigarettes per day, cigarette dependence (24), menthol use, and number of past year quit attempts.

### Outcome measures

*Puff patterns:* Puff patterns were measured in the laboratory setting via an eTop topography device attached to the mouthpiece of the e-cigarette (25). Consistent with the goal of the topography intervention, the primary topography outcome of interest was puff duration. Topography was measured at each lab-based e-cigarette use assessment (V1, V2, and V3).

*Cigarette craving:* Cigarette craving was assessed using the Questionnaire of Smoking Urges–Brief (QSU–Brief) (26), a 10-item scale on which participants rate their craving severity. Items were measured from 1 (strongly disagree) to 7 (strongly agree), and a total score was calculated with higher scores indicating greater craving severity. Cigarette craving was measured immediately prior to (under CO-verified overnight nicotine deprivation) and following the ad libitum vaping sessions during each of the three lab-based e-cigarette use assessment visits, and change scores were calculated.

*Nicotine withdrawal:* Nicotine withdrawal severity was measured using the 8-item Minnesota Nicotine Withdrawal Scale (27). Each item was measured from 0 (none) to 4 (severe), and total scores were calculated, with higher scores indicating a greater severity of withdrawal symptoms. Nicotine withdrawal was measured prior to and immediately following the ad libitum vaping session at each of the three lab visits, and change scores were calculated.

*Nicotine delivery:* Blood nicotine levels were measured prior to and immediately following each ad libitum session to assess increases in blood nicotine levels resulting from e-cigarette use. Change scores were calculated to determine the change in nicotine levels from pre- to post-vaping. Blood samples were analyzed for plasma nicotine by the Ultra performance liquid chromatography tandem mass spectrometry (UPLC-MS) method and were validated based on the principles outlined in the ICH M10 guidance. Bioanalysis was completed by the Bioanalytical Lab team within the Clinical Pharmacology Shared Resource of the University of Kansas Cancer Center.

*Cigarette smoking:* Participants self-reported cigarettes per day via the 7-Day Timeline Follow Back (28) at screening and week 12. From this data, change scores (screening to week 12) were calculated.

*Switch trajectory*: At week 12, participants were classified into one of the four switch trajectories based on the report of e-cigarette smoking and cigarette use from the week 12 7-day Timeline Follow Back: (1) *Complete switch*, defined as no cigarette smoking in the past 7 days with e-cigarette use and biochemical verification (eCO < 6 ppm), (2) *dual use with ≥ 50% CPD reduction*, defined as at least 50% reduction in CPD from screening and any e-cigarette use, (3) *dual use with < 50% CPD reduction*, defined as less than 50% reduction in CPD from screening and any e-cigarette use, and (4) *no switch*, defined as any smoking and no e-cigarette use.

## Data analysis

Descriptive statistics were presented for participant characteristics, smoking characteristics, and all outcomes for the overall sample and by treatment condition. Continuous variables were summarized using means and standard deviations, and categorical variables were summarized with frequencies and percentages. Changes in outcome measures of interest were calculated between follow-up visits (V2 or V3) and V1. As the low- and high-intensity training conditions did not differ during the acute phase (V1 to V2), means and standard deviations for these two conditions were pooled for acute outcomes, including change in cigarette craving, nicotine withdrawal, and nicotine delivery from V1 to V2. Due to the pilot nature of the study, the focus was not on statistical testing, but rather on effect size estimates. For this reason, to determine the impact of treatment condition on outcomes, effect sizes (i.e., Hedge’s g) were generated in place of *p*-values. For comparison between the three conditions from V1 to V3, the effect size reported was for the two groups with the largest difference between the change scores. Analyses were conducted using SAS 9.4. Unless otherwise noted, all analyses were among completers only.

## Results

*Participant characteristics:* Participants (*N* = 46) had a mean age of 62.3 years (SD = 9.4), 44.4% (*n* = 20) were male, 71.1% (*n* = 32) were white, 35.6% (*n* = 16) were married, a majority had at least a high school degree (*n* = 39; 86.7%), and owned a home (*n* = 26; 57.8%). Complete participant characteristics by study condition are included in Table 1.

**Table 1:** Baseline characteristics by study arm.

	Brief advice (*n* = 16) M (SD)	Low intensity training (*n* = 15*) M (SD)	High intensity training (*n* = 15) M (SD)	Overall (*N* = 46*) M (SD)
Age, years	62.0 (8.3)	59.6 (12.3)	65.1 (7.1)	62.3 (9.4)
Sex, male, *n* (%)	7 (43.8)	7 (50.0)	6 (40.0)	20 (44.4)
**Race^a^, *n* (%)**				
White	12 (75.0)	8 (57.1)	12 (80.0)	32 (71.1)
Black/African American	2 (12.5)	5 (35.7)	3 (20.0)	10 (22.2)
≥High school degree, *n* (%)	13 (81.3)	12 (85.7)	14 (93.3)	39 (86.7)
Annual income <$25,000, *n* (%)	6 (37.5)	9 (64.3)	10 (66.7)	25 (55.6)
Own home, *n* (%)	7 (43.8)	6 (42.9)	6 (40.0)	19 (42.2)
Married, *n* (%)	5 (31.3)	6 (42.9)	5 (33.3)	16 (35.6)
Cigarette dependence^b^	55.5 (15.2)	56.9 (13.6)	59.3 (12.9)	57.2 (13.7)
Baseline CPD	19.9 (9.2)	20.2 (11.0)	16.6 (11.7)	18.9 (10.5)
Menthol, *n* (%)	3 (18.8)	7 (50.0)	4 (26.7)	14 (31.1)
Past-year quit attempts, *n* (%)	8 (50.0)	5 (35.7)	9 (60.0)	22 (48.9)
Avg past-year quit attempts among those who reported any attempt	1.9 (1.1)	2.7 (1.5)	4.4 (4.5)	2.8 (2.5)

*One participant is missing all baseline demographic information. FTND, Fagerstrom Test for Nicotine Dependence. CPD, cigarettes per day. ^a^Two participants marked “some other race, ethnicity, or origin.” One participant preferred not to say.^b^Cigarette dependence was measured using a 16-item scale (24). Items were summed; greater scores indicate greater cigarette dependence. Possible range = 15–76.

*Baseline smoking characteristics*. Participants reported smoking an average of 18.9 cigarettes per day (SD = 10.5) and showed significant symptoms of dependence (M = 57.2; SD = 13.7); 31.1% (*n* = 14) reported menthol use. At baseline, 48.9% (*n* = 22) participants reported a past year 24-h quit attempt. Among those, participants reported an average of 2.8 (SD = 2.5) attempts. Baseline smoking characteristics by study condition are included in Table 1.

### Changes in outcomes from V1 to V2 (separated by ≥48 h)

*Puff duration:* Puff duration showed a very slight numerical decrease from V1 to V2 for brief advice (M∆ = −0.04 s; SD = 0.71) and a slight numerical increase for the combined low- and high-intensity training conditions (M∆ = 0.10 s; SD = 0.75). However, the between-group differences were minimal, resulting in a small effect of training (Hedge’s g = 0.20).

*Craving and withdrawal:* Complete data on V1 to V2 changes in craving and withdrawal are included in Table 2. Participants showed decreased reduction in within-session craving from V1 to V2 for both brief advice (M∆ = 0.36; SD = 1.81) and combined low- and high-intensity training (M∆ = 0.06; SD = 1.49), resulting in minimal difference between the two conditions (Hedge’s g = 0.19). Participants showed a numerical reduction in withdrawal from V1 to V2 for both brief advice (M∆ = −0.85; SD = 4.86) and combined low- and high-intensity training conditions (M∆ = −0.70; SD = 5.19). Numerical between-group differences were minimal (Hedge’s g = 0.03).

**Table 2:** Acute (≥48-h) and longer term (12 week) changes in puff duration, craving, withdrawal, and nicotine delivery by treatment condition.

		Human lab assessment visit 1 M (SD)	Human lab assessment visit 2 M (SD)	Change score (visit 1 to 2) M (SD)	Effect size	Human lab assessment visit 3 (Week 12) M (SD)	Change score (visit 1 to 3) M (SD)	Effect size^1^
Acute changes (≥48 h)								
Puff duration	Brief Advice	1.48 (0.93)	1.44 (0.79)	−0.04 (0.71)	0.20	---	---	---
	Low- + high-intensity training	1.58 (0.99)	1.68 (0.10)	0.10 (0.75)		---	---	---
Cigarette craving, within-session change^2^	Brief Advice	−1.32 (1.32)	−0.96 (1.42)	0.36 (1.81)	0.19	---	---	---
	Low- + high-intensity training	−1.26 (1.68)	−1.20 (1.72)	0.06 (1.49)		---	---	---
Nicotine withdrawal, within-session change^2^	Brief Advice	−3.00 (3.49)	−3.85 (3.44)	−0.85 (4.86)	0.03	---	---	---
	Low- + high-intensity training	−2.77 (4.52)	−3.47 (4.77)	−0.70 (5.19)		---	---	---
Nicotine delivery, ng/ml, within session change^2^	Brief advice	2.98 (4.85)	2.92 (8.18)	−0.06 (10.44)	0.08	---	---	---
	Low- + high-intensity training	2.99 (5.70)	2.36 (5.53)	−0.63 (4.41)		---	---	---
Long term changes over 12 weeks								
Puff duration	Brief Advice	1.56 (0.99)	---	---	---	1.24 (0.87)	−0.32 (0.56)	0.34
	Low-intensity training	1.44 (0.76)	---	---	---	1.33 (0.99)	−0.12 (1.23)	
	High-intensity training	1.88 (1.29)	---	---	---	1.39 (0.91)	−0.49 (0.95)	
Cigarette craving, within-session change^2^	Brief advice	−1.41 (1.39)	---	---	---	−0.77 (1.43)	0.64 (1.70)	0.44
	Low-intensity training	−0.74 (1.22)	---	---	---	−0.55 (0.80)	0.19 (1.25)	
	High-intensity training	−1.64 (1.77)	---	---	---	−0.78 (0.85)	0.87 (1.77)	
Nicotine withdrawal, within-session change^2^	Brief Advice	−2.82 (2.68)	---	---	---	−4.09 (3.59)	−1.27 (3.13)	0.46
	Low-intensity training	−1.58 (3.60)	---	---	---	−1.50 (2.94)	0.08 (4.62)	
	High-intensity training	−2.33 (4.01)	---	---	---	−4.08 (4.54)	−1.75 (3.22)	
Nicotine delivery, ng/mL, within-session change^2^	Brief Advice	3.93 (5.17)	---	---	---	5.03 (6.82)	1.10 (5.24)	0.56
	Low-intensity training	2.74 (5.47)	---	---	---	5.37 (7.94)	2.62 (7.44)	
	High-intensity training	3.91 (5.79)	---	---	---	3.34 (4.10)	−0.57 (3.15)	
Cigarette smoking, Cigarettes per day	Brief Advice	21.0 (8.3)	---	---	---	3.5 (4.9)	−17.6 (10.0)	0.78
	Low-intensity training	21.9 (11.7)	---	---	---	6.3 (5.5)	−15.6 (10.8)	
	High-intensity training	16.0 (11.7)	---	---	---	5.7 (6.1)	−10.3 (8.7)	

Change scores were calculated for the subset of participants who had values at both baseline and follow-up. SD, standard deviation. ^1^Hedge’s g for the largest difference between groups. ^2^Outcomes measured pre-session (under nicotine deprivation) and post-session (following ad libitum use), and change score calculated by subtracting pre from post measure.

*Nicotine delivery:* Data on V1 to V2 changes in nicotine delivery are included in Table 2. Counter to hypotheses, both brief advice (M∆ = −0.06 ng/mL; SD = 10.44) and the combined low- and high-intensity training conditions (M∆ = −0.63 ng/mL; SD = 4.41) showed reduced nicotine delivery at V2 relative to V1 with minimal between-group differences (Hedge’s g = 0.08).

### Changes in outcomes from V1 to V3 (separated by 12 weeks)

*Puff duration:* Complete V1 to V3 puff duration data are included in Table 2. Counter to hypotheses, across all treatment conditions, puff duration showed a slight numerical decrease from V1 to V3 (M∆_brief advice_ = −0.32 s; SD = 0.567, M∆l_ow intensity_ = −0.12 s; SD = 1.23, M∆_high intensity_ = −0.49 s; SD = 0.95; Hedge’s g = 0.34). Participant-level puff duration data from V1 to V3 are included in Supplementary Figure 1.

*Craving and withdrawal:* Counter to hypotheses, all conditions showed numerically greater reductions in craving with the use of e-cigarettes at V1 relative to V3. Between-group differences were minimal, resulting in a small effect of training on craving (Hedge’s g = 0.44). In terms of changes in withdrawal, brief advice and high-intensity training conditions showed numerically greater reductions of withdrawal symptoms with the use of e-cigarettes at V3 relative to V1(M∆_brief advice_ = −1.27; SD = 3.13, M∆_high intensity_ = −1.75; SD = 3.22), while the low intensity training condition showed numerically greater reductions at V3 relative to V1 (M∆_low intensity_ = 0.08; SD = 4.62). Between-group differences were minimal, resulting in a small effect of training condition (Hedge’s g = 0.46). Data on changes in craving and withdrawal from V1 to V3 are included in Table 2.

*Nicotine delivery:* Brief advice (M∆ = 1.10 ng/mL; SD = 5.24) and low-intensity training (M∆ = 2.62 ng/mL; SD = 7.44) both showed slight numerical increases in nicotine delivery from V1 to V3 with high-intensity training showing a slight numerical decrease (M∆ = −0.57 ng/mL; SD = 3.25; Hedge’s g = 0.56). Data on changes in nicotine delivery from V1 to V3 are included in Table 2.

*Cigarette smoking:* All conditions showed reductions in cigarettes per day. Specifically, the brief advice condition showed the numerically largest reduction (M∆ = −17.6; SD = 10.0), followed by low-intensity training (M∆ = −15.6; SD = 10.8) and high-intensity training (M∆ = −10.3; SD = 8.7; Hedges’ g = 0.78). See Table 2 for complete data.

*Switch trajectory:* Overall, 38.5% (*n* = 5) in the brief advice condition achieved a biochemically confirmed complete switch, followed by 25% (*n* = 3) in the high-intensity training condition and 9.1% (*n* = 1) in the low-intensity training condition. See Table 3 for complete switch trajectory data overall and by condition.

**Table 3:** Switch trajectory at week 12 by treatment condition.

	Brief advice (*n* = 13)	Low intensity training (*n* = 11)	High intensity training (*n* = 12)	Total (*N* = 36)
	*n* (%)	*n* (%)	*n* (%)	*n* (%)
Complete switch* (no smoking; +/− EC use; CO < 6 ppm)	5 (38.5)	1 (9.1)	3 (25.0)	9 (25.0)
Dual use with ≥ 50% CPD reduction *(≥ 50% reduction in smoking + any EC use)*	6 (46.2)	6 (54.6)	3 (25.0)	15 (41.7)
Dual use with < 50% CPD reduction *(< 50% reduction in smoking + any EC use)*	2 (15.4)	3 (27.3)	5 (41.7)	10 (27.8)
No switch *(smoking + no EC use)*	0 (0.0)	1 (9.1)	1 (8.3)	2 (5.6)

*One participant reported non-use of combustible and electronic cigarettes at follow-up and was classified as a complete switch. EC, electronic cigarette.

## Discussion

E-cigarettes are effective for smoking cessation (14) and are a particularly promising harm reduction tool for those who are unwilling or unable to quit smoking using standard cessation methods (11). COPD patients who smoke but have failed to quit and are unwilling to make another pharmacotherapy-assisted quit attempt may benefit from switching from combustible cigarettes to e-cigarettes. Interventions to enhance success with switching are understudied, particularly among COPD patients who smoke. Despite minimal differences between treatment groups, 25% of participants made a complete switch to e-cigarettes, and an additional 42% reduced their cigarette smoking by at least half. Consistent with these findings, all treatment conditions showed significant reductions in cigarettes per day from baseline to week 12. These findings are particularly promising given that these are individuals who would otherwise continue smoking cigarettes, due to an unwillingness to make another pharmacotherapy-assisted quit attempt in the next month. While the findings indicate that puff topography training does not enhance switch rates or smoking reduction beyond simple brief advice, it suggests that e-cigarettes hold significant promise in helping patients with COPD reduce and quit smoking.
Surprisingly, targeting topography did not effectively alter puff patterns to facilitate switching. This contrasts with observational studies showing that as individuals successfully switch to e-cigarette use, puff patterns change (i.e., puff duration lengthens while the number of puffs remains consistent) (17, 18). In fact, the pattern of results in the current study suggests that brief advice, the least intensive intervention, showed the greatest benefit across many outcomes, followed by low-intensity and high-intensity training. Of note, these observational studies were conducted among non-COPD patients. It is possible that for patients with lung disease who have no or limited experience with e-cigarettes, puffing like an experienced user from the point of initiation is simply too intense, given their disease and the differences between e-cigarette aerosol and cigarette smoke. Another potential explanation is that the amount of training was either too little or too much. For example, the training may be too brief to change puffing patterns that have been reinforced for many years. Alternatively, there may be a point of diminished return in which individuals receive too much intervention and experience treatment fatigue, putting them at high risk of continued smoking (29). Treatment fatigue was not directly assessed in the current study and remains an important area for continued study.
The current study was limited due to its pilot nature, which resulted in a small sample size and precluded formal significance testing. The study also lacked a proper control condition, and some measures were imprecise and prone to large variability, a particular challenge within this small sample size. While overall attrition was minimal, issues with the tolerability of the e-cigarette did arise, suggesting that tolerability may need to be addressed among COPD patients who smoke and are interested in transitioning to an e-cigarette.
In conclusion, despite the minimal impact of topography training, e-cigarettes may result in harm reduction for COPD patients who smoke, particularly those who have tried and failed to quit and would otherwise continue smoking cigarettes. To determine whether e-cigarettes offer benefits as an alternative to FDA-approved cessation medications, studies are needed to compare e-cigarettes with pharmacotherapy among COPD patients who smoke.

## Data Availability

The raw data supporting the conclusions of this article will be made available by the authors. However the author’s institution will require review of any requests for data, and access is therefore not immediately guaranteed.
