# Correction: E-cigarette puff topography instruction to enhance switching among COPD patients who smoke

**DOI:** 10.3389/fpubh.2025.1720297

**Published:** 2025-11-17

**Authors:** Eleanor L. S. Leavens, Theodore L. Wagener, Leah Lambart, Matthew S. Mayo, Lexie Brown, Edward F. Ellerbeck, Sandra A. Billinger, Branden Comfort, Jennifer Woodward, Brent Sear, Spencer Beaman, Lisa Sanderson Cox, Nicole L. Nollen

**Affiliations:** 1 Department of Population Health, University of Kansas School of Medicine, Kansas City, KS, United States; 2 University of Kansas Comprehensive Cancer Center, Kansas City, KS, United States; 3 Department of Internal Medicine, The Ohio State University, Columbus, OH, United States; 4 The Ohio State University Comprehensive Cancer Center, Columbus, OH, United States; 5 Department of Biostatistics and Data Science, University of Kansas Medical Center, Kansas City, KS, United States; 6 Department of Neurology, University of Kansas Medical Center, Kansas City, KS, United States; 7 Department of Internal Medicine, Division of General and Hospital Medicine, University of Kansas Medical Center, Kansas City, KS, United States; 8 Department of Family Medicine, Family Medicine and Community Health, University of Kansas Medical Center, Kansas City, KS, United States; 9 Clinical Pharmacology Shared Resource, University of Kansas Cancer Center, Kansas City, KS, United States

**Keywords:** smoking, COPD, tobacco harm reduction, topography, e-cigarette

There was a mistake in Table 1 as published. The data on cigarette dependence is incorrect. The corrected Table 1 appears below.

**Table 1:** Baseline characteristics by study arm.

	**Brief Advice (*n* = 16), M (SD)**	Low intensity training (*n* = 15^*^), M (SD)	**High intensity training (*n* = 15), M (SD)**	Overall (*N* = 46^*^), M (SD)
Age, years	62.0 (8.3)	59.6 (12.3)	65.1 (7.1)	62.3 (9.4)
Sex, male, *n* (%)	7 (43.8)	7 (50.0)	6 (40.0)	20 (44.4)
**Race**^a^, ***n*** **(%)**				
White	12 (75.0)	8 (57.1)	12 (80.0)	32 (71.1)
Black/African American	2 (12.5)	5 (35.7)	3 (20.0)	10 (22.2)
≥ High school degree, *n* (%)	13 (81.3)	12 (85.7)	14 (93.3)	39 (86.7)
Annual income < $25,000, *n* (%)	6 (37.5)	9 (64.3)	10 (66.7)	25 (55.6)
Own home, *n* (%)	7 (43.8)	6 (42.9)	6 (40.0)	19 (42.2)
Married, *n* (%)	5 (31.3)	6 (42.9)	5 (33.3)	16 (35.6)
Cigarette dependence^b^	55.5 (15.2)	56.9 (13.6)	59.3 (12.9)	57.2 (13.7)
Baseline CPD	19.9 (9.2)	20.2 (11.0)	16.6 (11.7)	18.9 (10.5)
Menthol, *n* (%)	3 (18.8)	7 (50.0)	4 (26.7)	14 (31.1)
Past year quit attempts, *n* (%)	8 (50.0)	5 (35.7)	9 (60.0)	22 (48.9)
Avg past year quit attempts among those that reported any attempt	1.9 (1.1)	2.7 (1.5)	4.4 (4.5)	2.8 (2.5)

^*^One participant missing all baseline demographic information. CPD, cigarettes per day.

^a^Two participants marked “some other race, ethnicity, or origin”. One participant preferred not to say.

^b^Cigarette dependence was measured using a 16-item scale (24). Items were summed; greater scores indicate greater cigarette dependence. Possible range = 15–76.

The measure of cigarette dependence was listed incorrectly within the **Methods** Section. A correction has been made to the section **Methods**, *Measures, Baseline smoking characteristics*.

The sentence previously read:

“*Baseline smoking characteristics:* Baseline smoking history included cigarettes per day, and cigarette dependence was measured via the Fagerstrom Test for Nicotine Dependence (24), menthol use, and the number of past-year quit attempts.”

The corrected sentence reads: “*Baseline smoking characteristics*. Baseline smoking history included cigarettes per day, cigarette dependence (24), menthol use, and number of past year quit attempts.”

The data associated with baseline cigarette dependence was incorrect. A correction has been made to Section **Results**, *Baseline smoking characteristics*.

The paragraph previously read:

“*Baseline smoking characteristics:* Participants reported smoking an average of 18.9 cigarettes per day (SD = 10.5) and had low-to-moderate cigarette dependence (M = 3.8; SD = 0.9); 31.1% (*n* = 14) reported menthol use. At baseline, 48.9% (*n* = 22) of participants reported a past-year 24-h quit attempt. Among those, participants reported an average of 2.8 (SD = 2.5) attempts. Baseline smoking characteristics by study condition are included in Table 1.”

The corrected paragraph reads:

“*Baseline smoking characteristics*. Participants reported smoking an average of 18.9 cigarettes per day (SD = 10.5) and showed significant symptoms of dependence (M = 57.2; SD = 13.7); 31.1% (*n* = 14) reported menthol use. At baseline, 48.9% (*n* = 22) participants reported a past year 24-h quit attempt. Among those, participants reported an average of 2.8 (SD = 2.5) attempts. Baseline smoking characteristics by study condition are included in Table 1.”

Citation number 24, corresponding to the measure of cigarette dependence, is incorrect. A correction has been made to the References. The correct reference details appear below:

“Strong DR, Pearson J, Ehlke S, Kirchner T, Abrams D, Tyalor K, et al. Indicators of dependence for different types of tobacco product users: descriptive findings from Wave 1 (2013–2014) of the Population Assessment of Tobacco and Health (PATH) study. *Drug Alcoh Depend*. (2017) 178:257–66. doi: 10.1016/j.drugalcdep.2017.05.010.”

The original version of this article has been updated.

