# Supplementary material for: E-cigarette puff topography instruction to enhance switching among COPD patients who smoke
Source: Front Public Health. 2025 Oct 2;13:1664400. doi: 10.3389/fpubh.2025.1664400 (PMC12528082; doi:10.3389/fpubh.2025.1664400)
Supplement: Supplementary file 1 [file Table_1.docx]

| Supplemental Table 1. Descriptives for craving, withdrawal, and nicotine measures pre- and post-vaping by study visit and treatment condition | | | | |
| --- | --- | --- | --- | --- |
|  |  | Visit 1 Pre-vaping  M (SD) | Visit 2  Pre-vaping  M (SD) | Visit 3  Pre-vaping  M (SD) |
| Cigarette craving | Brief Advice | 3.29 (1.85) | 2.76 (1.85) | 2.06 (1.71) |
|  | Low Intensity Training | 3.82 (1.84) | 2.93 (1.77) | 2.30 (1.29) |
|  | High Intensity Training | 3.32 (1.84) | 2.93 (1.89) | 2.70 (1.44) |
| Nicotine withdrawal | Brief Advice | 5.79 (4.46) | 6.50 (4.83) | 6.33 (5.28) |
|  | Low Intensity Training | 9.47 (7.12) | 8.20 (7.82) | 5.08 (3.23) |
|  | High Intensity Training | 8.00 (6.61) | 7.67 (6.86) | 9.67 (6.30) |
| Serum nicotine, ng/mL | Brief Advice | 6.75 (3.31) | 7.64 (4.62) | 10.02 (6.10) |
|  | Low Intensity Training | 5.51 (4.54) | 6.92 (3.81) | 6.69 (2.84) |
|  | High Intensity Training | 5.17 (3.41) | 6.57 (4.72) | 5.51 (2.50) |
| *Note.* Data are from participants with complete data at pre-vaping for each visit. | | | | |
